# Identification of *Staphylococcus aureus* Cellular Pathways Affected by the Stilbenoid Lead Drug SK-03-92 Using a Microarray

**DOI:** 10.3390/antibiotics6030017

**Published:** 2017-09-11

**Authors:** William R. Schwan, Rebecca Polanowski, Paul M. Dunman, Sara Medina-Bielski, Michelle Lane, Marc Rott, Lauren Lipker, Amy Wescott, Aaron Monte, James M. Cook, Douglas D. Baumann, V.V.N. Phani Babu Tiruveedhula, Christopher M. Witzigmann, Cassandra Mikel, Md Toufiqur Rahman

**Affiliations:** 1 Department of Microbiology, University of Wisconsin-La Crosse, La Crosse, WI 54601, USA; rpolanowski@uwlax.edu (R.P.); s.medinabielski@gmail.com (S.M.-B.); lanem7285@gmail.com (M.L.); mrott@uwlax.edu (M.R.); lipker.laur@uwlax.edu (L.L.); amywescott16@gmail.com (A.W.); cassandra.mm010@gmail.com (C.M.); 2 Emerging Technology Center for Pharmaceutical Development, University of Wisconsin-La Crosse, La Crosse, WI 54601, USA; amonte@uwlax.edu; 3 School of Medicine and Dentistry, University of Rochester, Rochester, NY 14642, USA; paul_dunman@urmc.rochester.edu; 4 Department of Chemistry and Biochemistry, University of Wisconsin-La Crosse, La Crosse, WI 54601, USA; 5 Department of Chemistry and Biochemistry, University of Wisconsin-Milwaukee, Milwaukee, WI 53211, USA; capncook@uwm.edu (J.M.C.); tiruvee2@uwm.edu (V.V.N.P.B.T.); witzigm2@uwm.edu (C.M.W.); mdrahman@uwm.edu (M.T.R.); 6 Department of Mathematics and Statistics, University of Wisconsin-La Crosse, La Crosse, WI 54601, USA; dbaumann@uwlax.edu

**Keywords:** stilbene, microarray, *Staphylococcus aureus*, gene regulation, drug mechanism of action, sortase, biofilm

## Abstract

The mechanism of action for a new lead stilbene compound coded SK-03-92 with bactericidal activity against methicillin-resistant *Staphylococcus aureus* (MRSA) is unknown. To gain insight into the killing process, transcriptional profiling was performed on SK-03-92 treated vs. untreated *S. aureus*. Fourteen genes were upregulated and 38 genes downregulated by SK-03-92 treatment. Genes involved in sortase A production, protein metabolism, and transcriptional regulation were upregulated, whereas genes encoding transporters, purine synthesis proteins, and a putative two-component system (SACOL2360 (MW2284) and SACOL2361 (MW2285)) were downregulated by SK-03-92 treatment. Quantitative real-time polymerase chain reaction analyses validated upregulation of *srtA* and *tdk* as well as downregulation of the MW2284/MW2285 and purine biosynthesis genes in the drug-treated population. A quantitative real-time polymerase chain reaction analysis of *MW2284* and *MW2285* mutants compared to wild-type cells demonstrated that the *srtA* gene was upregulated by both putative two-component regulatory gene mutants compared to the wild-type strain. Using a transcription profiling technique, we have identified several cellular pathways regulated by SK-03-92 treatment, including a putative two-component system that may regulate *srtA* and other genes that could be tied to the SK-03-92 mechanism of action, biofilm formation, and drug persisters.

## 1. Introduction

*Staphylococcus aureus* is a common inhabitant of the human body that also causes numerous infections, including skin and soft tissue infections as well as more serious infections, such as pneumonia and bacteremia [1]. Presently, around 60% of *S. aureus* clinical isolates are methicillin-resistant *S. aureus* (MRSA) [2], and this bacterium is a leading cause of nosocomial infections in the United States [3,4]. In 1997, community-associated methicillin-resistant *S. aureus* (CA-MRSA) strains emerged in the United States, causing infections in younger people, including necrotizing pneumonia [5,6,7]. Although skin infections caused by CA-MRSA are still prevalent, invasive MRSA infections have decreased [3,8]. In addition to methicillin resistance, CA-MRSA strains are becoming multidrug resistant at an alarming rate [9,10,11]. Heterogeneous vancomycin-intermediate *S. aureus* and vancomycin-resistant strains of *S. aureus* have led to vancomycin being less effective against some *S. aureus* infections [12,13,14,15]. Tolerance to vancomycin now has been reported to be as low as 3% and as high as 47% [16,17]. New drugs are needed to treat MRSA infections; however, most drugs currently in development are derivatives of drugs already being marketed [18,19]. *S. aureus* is one of the ESKAPE pathogens (*Enterococcus faecium*, *Staphylococcus aureus*, *Klebsiella pneumoniae*, *Acinetobacter baumannii*, *Pseudomonas aeruginosa* and *Enterobacter* species) targeted by the 10 × 20 initiative to develop 10 new, safe and effective antibiotics approved by 2020 [20].

In support of the 10 × 20 initiative, a new antibiotic identified as (E)-3-hydroxy-5-methoxystilbene with promising activity against *S. aureus* was identified from *Comptonia peregrina* (L.) Coulter (“sweet fern”) [21]. A structure–activity relationship analysis identified our lead compound, (E)-3-(2-(benzo[b]thiophen-2-yl)vinyl)-5-methoxyphenol; for simplicity, SK-03-92. SK-03-92 was rapidly bactericidal (killing 90% of the population within an hour) against every Gram-positive species that was tested, including MRSA strains [22]. Importantly, a combined safety and pharmacokinetic study demonstrated that the SK-03-92 lead drug was safe in mice [23]. As with all antimicrobials, therapeutic treatment can result in residual bacteria not being killed by that antimicrobial, a phenomenon known as persistence [24,25,26]. Drug persisters are phenotypically different than the parent strain, but are not true drug resistant variants because the MICs of the drug persisters are the same as their parent strains [27,28]. Persisters are thought to be a major component of bacterial biofilms, allowing significant drug tolerance [29,30]. Many drugs used to treat *S. aureus* infections have drug persister population emerge that are recalcitrant to treatment. To gain insight into the mechanism of action of SK-03-92 and the mechanism of *S. aureus* persistence to SK-03-92 treatment, the effect of SK-03-92 on *S. aureus* cells was assessed by transcriptional profiling in the *S. aureus* strain MW2.

## 2. Results and Discussion

### 2.1. General Transcriptome Response of SK-03-92 Treatment

New drugs to treat *S. aureus* infections are urgently needed, and SK-03-92 holds considerable promise. SK-03-92 has a stilbenoid backbone [22] and is bactericidal within an hour; however, 10% of the population survives as drug persisters that can grow in media containing up to 32 μg/mL of SK-03-92 but with an MIC equivalent to untreated *S. aureus* cells. The mechanism of action for SK-03-92 is unknown. To ascertain the effects of SK-03-92 treatment on the transcriptome of *S. aureus*, total RNA was isolated from *S. aureus* strain MW2 cultures (Table 1) treated for 30 min with 8× the MIC of SK-03-92 and untreated MW2 cultures and an RNA microarray was performed. A total of 52 genes were dysregulated by the SK-03-92 drug treatment (Table 2), representing 2% of the total *S. aureus* transcriptome. This is remarkable because transcriptional profiling of other bactericidal compounds has shown a larger effect on the *S. aureus* transcriptome, including ortho-phenylphenol (24%) [31], amicoumacin A (20%) [32] and daptomycin (5% to 32%) [33,34]. Interestingly, the number of downregulated genes (73.1%) greatly surpassed the number of upregulated genes (26.9%).

An examination of genes affected by treatment with other stilbene type compounds demonstrates the disparity in their transcript profile compared to treatment with SK-03-92. Pterostilbene, another stilbenoid compound, in *Saccharomyces cerevisiae* showed 1189 genes that were dysregulated: 1007 upregulated (85%) and 182 downregulated (15%) [35]. Microarray analysis with resveratrol treated *Schizosaccharomyces pombe* showed 480 genes dysregulated, 377 genes that were upregulated and 103 that were downregulated [36]. RNA sequence analysis of resveratrol treated *S. aureus* cells demonstrated 444 dysregulated genes, 201 upregulated and 243 downregulated [37]. The majority of the genes in our study had a two- to four-fold difference in transcript abundance when comparing SK-03-92 treated vs. untreated *S. aureus* cultures. Very few genes dysregulated by SK-03-92 were previously shown to be dysregulated by resveratrol (e.g., downregulation of the *purD*, *purH*, *purL*, *lrgA*, and *sdhC* genes). Only three genes had a 10-fold or higher change in transcript levels, which included two genes annotated to be part of a putative two-component system (TCS) (*SACOL2360* (annotated as *MW2284* in MW2 strain) = 14.1-fold lower and *SACOL2361* (annotated as *MW2285* in MW2 strain) = 26.9-fold lower) as well as the *glpD* gene encoding glycerol-3-phosphate dehydrogenase (10-fold higher).

Dysregulated genes tied to a potential mechanism of action for SK-03-92 included *glpD*, *adhE* (*SACOL0135*), *adhP* (*SACOL0660*), and *sdhC* (*SACOL1158*). GlpD funnels electrons into the respiratory chain via quinone or menaquinone reduction coupled to the oxidation of glycerol-3-phosphate to glycerone phosphate (dihydroxyacetone phosphate) [40], which can be enzymatically or non-enzymatically transformed into methylglyoxal (MG) [41]. Higher concentrations of MG are thought to halt bacterial growth by damaging proteins by acting as a protein glycating agent that mainly affects arginine residues [42,43]. In *Candida albicans*, ADH1 catalyzes the NAD+ linked oxidation of MG to pyruvate and disruption of the *adh1* gene in *C. albicans* caused accumulation of MG followed by inhibition of growth [44]. The dysregulation of *glpD* and *adh* genes suggests that MG was accumulating and glycation was occurring in SK-03-92-treated *S. aureus*. MG glycation of proteins, lipids, and DNA generate advanced glycation end products (AGEs) [43]. Importantly, GlpD has been implicated in drug persistence in *Escherichia coli* [45] and *S. aureus* [34].

A number of genes involved in metabolism were also dysregulated by SK-03-92 treatment, including the *gcvH* gene that encodes GcvH, which shuttles the methylamine group of glycine from the P-protein to the T-protein via a lipoyl group [46]. Genes associated with protein degradation and repair had altered transcript abundance in SK-03-92-treated *S. aureus*. Transcripts encoding a putative repair system for deglycation of Amadori protein adducts derived from ribose-5-P (*ptpA*) [47] showed altered abundance in SK-03-92-treated *S. aureus*, as did the transcript encoding the enzyme that produces ribose-5-P (*SACOL2605*). The formation of Amadori protein adducts occurs spontaneously via a dehydrogenation mechanism when ribose-5-P interacts with an amine, such as the lysine residues of proteins. Amadori glycated proteins undergo further spontaneous reactions to become AGEs. AGEs promote protein aggregation [47,48]. Since *ptpA* transcript abundance was increased 2.3-fold and the kinase transcript *SACOL2605* was decreased 9.6-fold, ribulosamine substrates produced were likely not being deglycated, and protein repair was not occurring. Phase-dark and phase-bright inclusions were observed microscopically in SK-03-92-treated *B. subtilis*, consistent with perturbation of proteostasis resulting in visible accumulation of protein aggregates [49]. Uncontrolled protein aggregation is toxic to cells [48]. 

Three genes associated with purine synthesis were downregulated: *purD*, *purH*, and *purL*. Purine metabolism is a necessary part of DNA synthesis and energy production in *S. aureus* [50]. Genes involved in purine metabolism are often downregulated after treatment with a drug or plant extract [51,52,53]. In addition, one gene associated with pyrimidine synthesis, *tdk*, was upregulated. Thymidine kinase transfers the terminal phosphate from ATP to thymidine or deoxyuridine [54]. A decrease in the synthesis of purines coupled with an increase in phosphorylation of pyrimidines could result in a dramatic reorganization of the intracellular nucleotide pool. Moreover, less purine metabolism is often tied to drug persister populations [55,56]. Disruption of nucleotide metabolism in a library of *S. aureus* transposon insertion mutants caused a decrease in persister formation frequency when treated with rifampicin [57].

Consistent with the formation of persister strains, mRNA levels of genes linked to programmed cell death (PCD) were decreased in *S. aureus* cultures treated with SK-03-92. Specifically, the Cid/Lrg (holin/antiholin) system, which controls autolysis and affects the distribution of extracellular DNA in *S. aureus* during biofilm development [58,59,60]. This prokaryotic PCD is analogous to the bcl-2 pro-apoptotic effector and anti-apoptotic mediated apoptosis in eukaryotes [61,62].

Twelve putative transport genes were dysregulated encoding for proteins involved in anion transport, a cation efflux family protein, two phosphotransferase system (PTS) transporters, a sodium:alanine symporter, sodium:dicarboxylate symporter family protein, and a copper ion binding protein. The only true virulence factor genes affected by SK-03-92 treatment were the *SACOL0151 cap5P*, *epiE*, *SACOL2333* gene encoding a YnfA family protein putative transport small multidrug resistance family-3 protein [63], and the *srtA* gene encoding sortase A that will be described in more detail below [64]. Five genes identified by the microarray were annotated as hypothetical proteins with no known function (three downregulated and two upregulated).

### 2.2. Genes of a Putative TCS Are Significantly Downregulated by SK-03-92 Treatment

A surprising microarray result that was no known *S. aureus* global regulatory genes were shown to be affected by the drug treatment. Microarray analysis of daptomycin treated *S. aureus* demonstrated that the the *icaR* gene was dysregulated compared to untreated cells [34]. Our microarray showed that a *tetR*-family transcriptional regulator, SACOL2340, and two genes that comprise a putative TCS in *S. aureus* annotated as *MW2284* (14.1-fold downregulated) and *MW2285* in strain MW2 (26.3-fold downregulated) were downregulated. A bioinformatic analysis of the putative MW2284 and MW2285 proteins suggest that they comprise a putative two-component regulatory system where MW2284 (LytTR superfamily regulator protein) is the response regulator protein and MW2285 (membrane protein) is the sensor kinase protein. MW2284 was identified as a 440-bp ORF encoding a putative 14.7-kDa transcriptional regulator protein and MW2285 was identified as a 455-bp ORF encoding a putative 15.1-kDa histidine kinase sensor protein. The *MW2285* ORF has a 3-bp overlap with the *MW2284* ORF. BLASTP, PSI-BLAST, and BLASTN bioinformatics analyses [65] showed that MW2284 aligned with other two-component regulatory system regulator proteins and MW2285 aligned with other two-component regulatory system sensor proteins. Both proteins have homology with LytTR superfamily proteins involved in the regulation of bacterial genes [66]. LytTR proteins regulate virulence gene expression in a variety of bacterial species including *S. aureus*. The AgrA transcriptional regulator is one of these LytTR-type proteins [67]. Moreover, the *MW2284* and *MW2285* ORFs appeared to be conserved across a wide number of Gram-positive species, including all *Staphylococcus* and *Streptococcus* species, as well as *Bacillus*, *Clostridium*, *Lactobacillus*, *Listeria*, and *Leuconostoc*.

The same LytTR TCS dysregulated in SK-03-92-treated *S. aureus* was upregulated in purine synthesis deficient mutants in *S. aureus* [68]. The putative sensor kinase (MW2285) was upregulated in *purH* mutants and the response regulator (MW2284) was upregulated in *purA* mutants (adenylosuccinate synthetase involved in purine biosynthesis). The response regulator component transcript was also upregulated during anaerobic growth in another study [69]. A transposon mutant of the sensor kinase component has been previously shown to be viable, capable of producing a more robust biofilm, and had a lower LD50 than the parent strain [70,71]. The mechanistic link between defects in purine synthesis, persister formation, and the LytTR regulatory system remains unclear. Furthermore, RNAseq analysis of resveratrol treated *S. aureus* cells showed an almost 8-fold downregulation of the *MW2284* gene, but no effect on the *MW2285* gene [37].

### 2.3. Validation of Microarray Data by qRT-PCR

**Figure 1:**
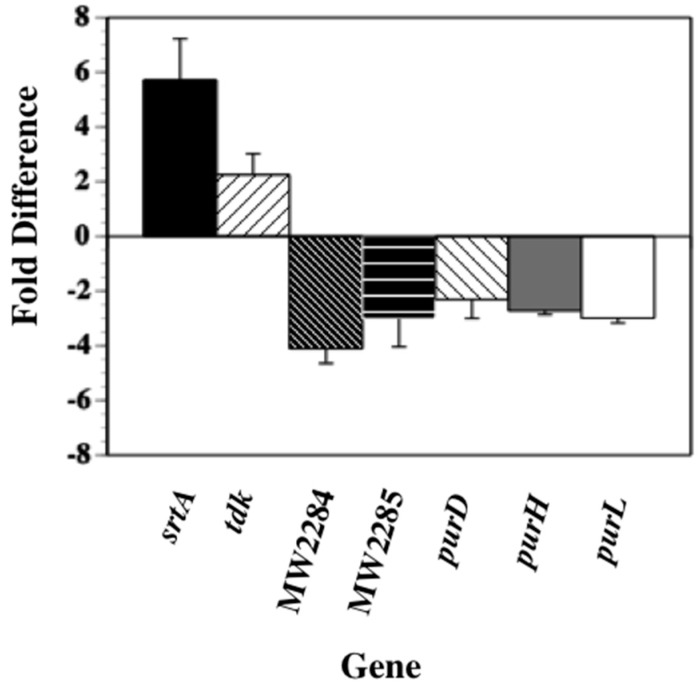
Quantitative reverse transcribed-polymerase chain reaction results of *S. aureus* MW2 cells treated with 8× the SK-03-92 MIC vs. untreated cells. The data represents the mean + standard deviation from at least three separate runs.

The microarray results were confirmed using qRT-PCR analyses on RNAs from 8× the MIC SK-03-92 treated MW2 cells vs. untreated MW2 cells. Transcription of the *srtA* gene was significantly upregulated almost 6-fold (*p* < 0.006, Figure 1) and the *tdk* gene was also upregulated 2.1-fold (*p <* 0.03) in SK-03-92 treated cells vs. untreated cells. On the other hand, several genes involved in purine biosynthesis (*purD*, *purH*, and *purL*) were shown to be significantly downregulated 2.2- to 2.4-fold (*p* < 0.01 to 0.04), whereas the *MW2284* and *MW2285* genes were downregulated 4- (*p* < 0.01) and 3-fold (*p* < 0.003), respectively, in the SK-03-92 treated samples. These results confirmed that treatment with the SK-03-92 lead compound caused dysregulation of the *srtA*, *tdk*, *purD*, *purH*, *purL*, *MW2284*, and *MW2285* genes.

### 2.4. SK-03-92 Treatment Causes Alteration of Nucleotide Pool

**Figure 2:**
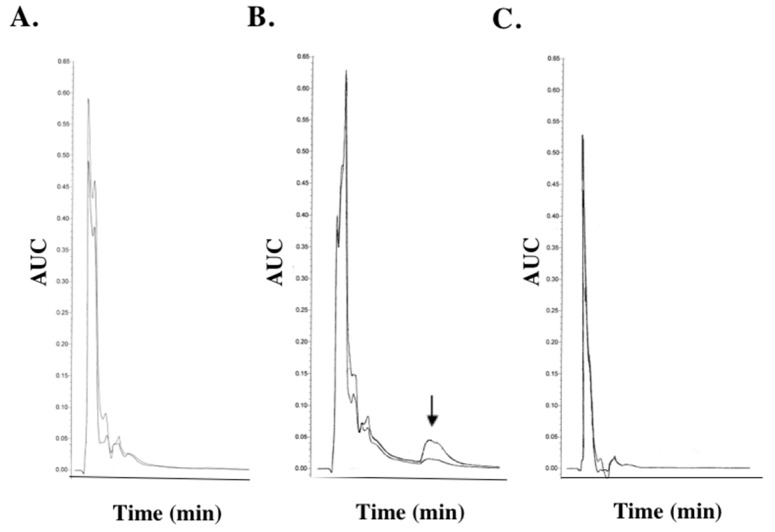
Absorbance (254 and 280 nm) of the formic acid extracted nucleotide pool of log-phase *S. aureus* ATCC 29213 after 20 min with either (**A**) no treatment, (**B**) 60 μg/mL mupirocin, or (**C**) 16 μg/mL SK-03-92. Arrow denotes the (p)ppGpp peak.

Because three *pur* genes involved in purine synthesis and the *tdk* gene were dysregulated by SK-03-92 treatment, the rapid accumulation of the bacterial alarmone (p)ppGpp and the state of the intracellular nucleotide pool were examined using high-performance liquid chromatography (HPLC, Waters, Milford, MA, USA). Inhibition of isoleucyl tRNA synthetase by mupirocin has been shown to induce production of (p)ppGpp in *S. aureus* [72,73]. A highly phosphorylated ribonucleotide, (p)ppGpp, can be identified via rapid separation of the *S. aureus* nucleotide pool using anion-exchange HPLC, where (p)ppGpp elutes as a late peak, which can be detected by absorbance at 254 and 280 nm [74]. In control experiments, this late peak was not detected in untreated cells (Figure 2A), but was detected following treatment with mupirocin (Figure 2B). No (p)ppGpp was detected following treatment with SK-03-92 (Figure 2C). However, the composition and quantity (area under curve) of the nucleotide pool was altered in SK-03-92 treated *S. aureus* as compared to untreated cells (Figure 2A vs. Figure 2C), suggesting that dysregulation of *tdk* and the three purine biosynthesis genes by SK-03-92 treatment depleted the nucleotide pool.

### 2.5. Biofilm Formation Increases as the Concentration of SK-03-92 Increases

**Figure 3:**
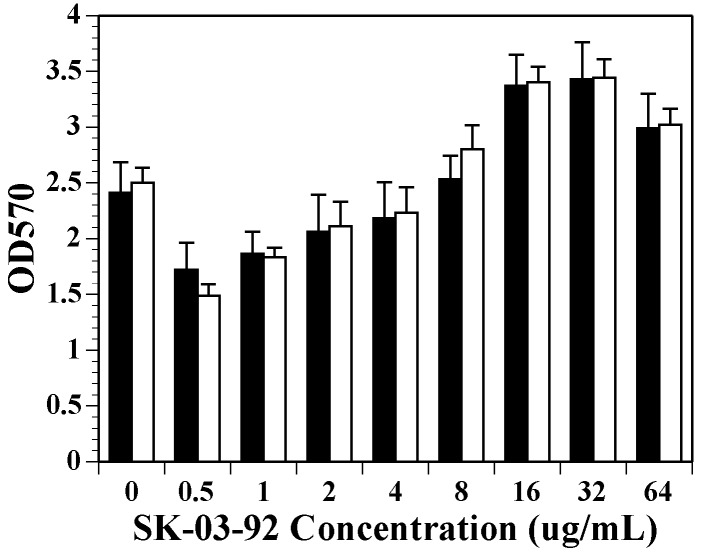
The effects of SK-03-92 drug concentration on 24 h biofilm formation (OD_570_) for *S. aureus* strains JE2 (black column) and MW2 (white column). All experiments represent the mean + standard deviation of at least 10 runs done in triplicate.

With an increase in *srtA* transcript abundance shown by the qRT-PCR results, an increase in biofilm formation would be expected following SK-03-92 treatment. To further analyze the effects of the increase in *srtA* transcription, a biofilm assay in microtiter plates was performed after SK-03-92 drug treatment (Figure 3). Wild-type JE2 and MW2 cultures were tested following SK-03-92 drug treatment (range 0.5–0.64 μg/mL). The JE2 culture grown without drug showed an OD_570_ of 2.41, whereas the MW2 culture had an OD_570_ of 2.50.

The SK-03-92 drug had a biphasic effect on the wild-type strains. At low concentrations, the drug reduced biofilm formation as exhibited by the 0.5 and 1 μg/mL data points that were significant for both strains that were tested (*p* < 0.05). As concentrations of SK-03-92 increased, the OD570 readings increased, plateauing at 32 μg/mL for both strains. Strain MW2 showed significant increases in biofilm formation going from 0 μg/mL to 8–64 μg/mL SK-03-92 concentration (*p* < 0.05). A similar finding was observed when *Candida* species grown as a biofilm were exposed to varying concentrations of echinocandin [75]. *Candida* treated with low drug concentrations killed the fungal cells but at concentrations higher than the MIC showed there was an increase in the cell density of the biofilms. Echinocandin acting on the *Candida* species has the same effect as our SK-03-92 drug, triggering upregulation of a specific gene that increases biofilm formation.

Under normal growth conditions, biofilm formation is not necessary for a cell, but under stressful environmental conditions, such as exposure to the SK-03-92 drug, biofilm formation would greatly benefit the *S. aureus* population. Formation of a biofilm would benefit cells by allowing for the formation of persister cell populations [76]. When biofilms form, the cells at the base of the biofilm slow or stop most cell metabolism and go into a dormant state, allowing the organisms to survive in the presence of a drug, for example SK-03-92. In addition, cells in a biofilm often undergo quorum sensing, which also can lead to the emergence of persister cells [77].

### 2.6. A Sortase A Mutant Has a Lower MIC against SK-03-92 Than Wild-Type

Since the putative MW2284/MW2285 TCS appears to repress transcription of the *srtA* gene, this regulatory effect could be tied to the mechanism of action of the SK-03-92 drug. Sortase A was first described in *S. aureus* in 1999 [64]. The protein covalently anchors surface proteins (e.g., fibronectin-binding protein, fibrinogen-binding protein, protein A, clumping factors, collagen adhesion protein) to the cell wall of *S. aureus* and other Gram-positive bacteria [77]. An LPXTG motif [78,79,80] is common among these anchored proteins and many are important for phase I of biofilm formation that allows attachment to biotic or abiotic surfaces [81]. A mutation of the *srtA* gene caused less expression of several cell wall anchored surface proteins [82,83]. Moreover, *srtA* mutants are attenuated compared to the wild-type strain in a variety of murine models of infection [82,84,85].

Because *srtA* and *MW2284/MW2285* transcription were affected by SK-03-92 treatment, MICs were performed using the SK-03-92 lead compound on an *srtA* mutant (NE1787), *srtB* mutant (control, NE1363), *MW2284* mutant (NE671), and *MW2285* mutant (NE272) compared to the wild-type strain JE2 [38]. The *srtB*, *MW2284*, and *MW2285* mutants had MICs that were equal to the wild-type strain (Table 3). However, the *srtA* mutant had an MIC that was 2-fold lower than the wild-type strain. When a *Listeria monocytogenes srtA* mutant was tested [39], the MIC for the *srtA* strain was 8-fold lower than the wild-type strain. A *L. monocytogenes srtB* mutant had the same MIC as the wild-type bacteria.

Presumably, SK-03-92 treatment causes downregulation of the *MW2285* gene with an effect that would be similar to a mutation in the MW2285 gene. The regulatory effect could be derepression of *srtA* transcription. Either event would create more SrtA protein that in turn would allow greater extracellular presentation of proteins on the surface of *S. aureus* cells. This result may suggest that something tethered to the cell walls by sortase A that is conserved in both species may be tied to the mechanism of action of the SK-03-92 drug, and we are exploring this possibility.

### 2.7. Mutations in the MW2284/MW2285 Two-Component Regulatory Genes Cause an Upregulation of the srtA Gene

**Figure 4:**
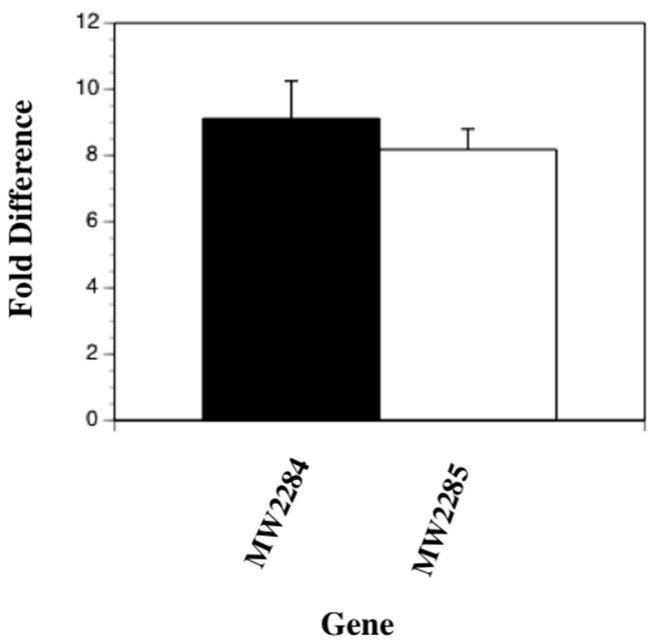
Quantitative reverse transcribed-polymerase chain reaction results of *S. aureus srtA* transcription in wild-type bacteria compared to MW2284 and MW2285 mutants. The data represents the mean + standard deviation from three separate runs.

Since the microarray results showed significant upregulation of the *srtA* gene and downregulation of the *MW2284* and *MW2285* genes, we hypothesized that the MW2284 gene product, a putative transcriptional regulator protein, may be repressing the *srtA* gene. To confirm that the putative two-component regulatory system (MW2284/MW2285) may be involved in repressing the *srtA* gene, we obtained transposon mutant strains from the Nebraska Transposon Mutant Library [38] with insertion mutations in the *MW2284* and *MW2285* genes. A qRT-PCR analysis was then undertaken on RNA isolated from the NE272 (*MW2285* mutation) and NE671 (*MW2284* mutation) strains compared to the wild-type strain JE2, targeting the *srtA* gene. The results showed that mutations in both the *MW2284* and *MW2285* genes led to a 9.2-fold (*p* < 0.005) and 8.1-fold (*p* < 0.0008) upregulation of *srtA* transcription, respectively, suggesting that this putative two-component regulatory system may be repressing transcription of the *srtA* gene (Figure 4).

## 3. Experimental Section

### 3.1. SK-03-92 Synthesis

SK-03-92 was synthesized as described previously [22].

### 3.2. Bacterial Strains and Growth Conditions

The *S. aureus* MW2 strain [7] used for the initial microarray and confirmatory qRT-PCRs (Table 1) was obtained from Jean Lee (Brigham and Young Hospital, Boston, MA, USA). *S. aureus* strains JE2 (wild-type), NE671 (MW2284), and NE272 (MW2285) were obtained from the Network on Antimicrobial Resistance in *Staphylococcus aureus* (NARSA) strain repository (Table 1), representing part of the Nebraska Transposon Mutant Library [38]. Strain JE2 is a plasmid-cured derivative of a USA300 CA-MRSA [86]. Phillip Klebba (Kansas State University, Manhattan, KS, USA) [39] provided the *Listeria monocytogenes* wild-type strain EGD as well as the isogenic *srtA* and *srtB* mutant strains. All strains were grown in brain heart infusion broth (Becton Dickinson, Franklin Lakes, NJ, USA) or trypticase soy broth (Becton Dickinson) shaken 250 rpm at 37 °C. The transposon mutant strains had 5 μg/mL of erythromycin (Sigma-Aldritch, St. Louis, MO, USA) added to the media.

### 3.3. RNA Extractions

Total RNA was isolated from *S. aureus* MW2 cells grown to exponential growth phase (OD_600_ approximately 0.5) either treated with dimethyl sulfoxide (DMSO) or 8× the MIC of SK-03-92 dissolved in DMSO using TRizol extraction (Life Technologies, Carlsbad, CA, USA) according to manufacturer′s instructions with an additional lysostaphin treatment step to help lyse the *S. aureus* cell walls. The RNA samples were digested with DNase I (New England Biolabs, Ipswich, MA, USA) followed by phenol and chloroform extractions to remove the protein. RNAs were run on 0.8% agarose gels to confirm concentration and integrities of the RNAs. To assess DNA contamination of the samples, PCRs were performed on the RNA samples using SaFtsZ1 and SaFtsZ2 primers (see Table 4). The PCR conditions for amplification with the SaFtsZ1/SaFtsZ2 primers was as follows: 94 °C, 1 min; 55 °C, 1 min; and 72 °C, 1 min for 35 cycles.

### 3.4. Microarray

Total RNAs from cells treated with DMSO or 8× the MIC of SK-03-92 were converted to cDNAs, biotinylated, and hybridized to *S. aureus* GeneChips following the manufacturer′s recommendations (Affymetrix, Santa Clara, CA, USA). Agilent GeneSpring G× 7.3 software (Santa Clara, CA, USA) was used to gauge transcript differences and a two-fold or higher difference in the transcript level for one population over the other was considered significant. Nucleic acid sequences with a ≥2-fold change in transcriptional abundance were mapped to the *S. aureus* COL genome (taxid: 93062) via BLASTN, BLASTX, or PSI-BLAST analysis [65] through the National Center for Biotechnology Information (NCBI, Bethesda, MD, USA) website and their putative products were annotated.

### 3.5. cDNA Synthesis

The cDNAs were synthesized from 5 μg of total RNA from SK-03-92 treated or untreated *S. aureus* MW2 using a First-Strand Synthesis kit (Life Technologies) according to manufacturer′s instructions.

### 3.6. Real Time-Quantitative Polymerase Chain Reaction (qRT-PCR)

All of the qRT-PCRs were performed using the LightCycler FastStart DNA MasterPLUS SYBR Green kit according to manufacturer′s instructions (Roche, Indianapolis, IN, USA). Primers used in this study were based off of the MW2 sequenced genome [87] and synthesized by Integrated DNA Technologies (Coralville, IA, USA) that are shown in Table 4. A LightCycler 1.5 machine (Roche) or a CFX96 machine (BioRad, Hercules, CA, USA) were used throughout the study. The *guaB* and *ftsZ* housekeeping genes were used as standardization controls. Each RT-qPCR run followed the minimum information for publication of quantitative real-time PCR experiments guidelines [88]. The qRT-PCRs were done at least three times under the following conditions: 94 °C, 20 s; 55 °C, 30 s; and 72 °C, 1 min for 35 cycles. The level of target gene transcripts in MW2 cells was compared to the *guaB* and *ftsZ* genes. Crossover points for all genes were standardized to the crossover points for *ftsZ* and *guaB* in each sample using the 2^−ΔΔCT^ formula [89].

### 3.7. HPLC

High-performance liquid chromatography was used to detect the presence of (p)ppGpp in the intracellular nucleotide pools of mid-log phase *S. aureus* cells following treatment with SK-03-92, mupirocin (positive control), or dimethyl sulfoxide (negative control) [90,91,92]. SK-03-92 was added at 16 µg/mL to 100 mL mid-log (OD_600_ = 0.4–0.6) culture in cation-adjusted Mueller Hinton broth and incubated for 20 min with shaking at 37 °C. Mupirocin was added at 60 µg/mL. Cells were collected after a 20 min incubation by centrifugation at 10,000× *g* for 10 min at 4 °C. The supernatant was discarded and the cell pellet suspended in 12 mL of ice cold 0.4 M formic acid (pH 3.5). After 30 min on ice, the cell extract was centrifuged at 10,000× *g* for 10 min at 4 °C to remove cell debris. The supernatant was evaporated under vacuum and filtered (0.2 µm pore size). Filtered cell extract was stored at −20 °C until use. Fifty microliters of filtered cell extract were loaded on a Hypersil SAX column (Thermo Fisher Scientific, Waltham, MA, USA) (5 µm, 4.6 × 250 mm) at a flow rate of 1.0 mL/min in 0.45 M potassium phosphate 0.05 M magnesium sulfate buffer (pH 3.5) using a Waters 600E pump and 996 photodiode array detector (Waters, Milford, MA, USA). Absorbance of the separated nucleotide pool was monitored at 254 and 280 nm.

### 3.8. Biofilm Assay

To determine the effect of SK-03-92 treatment on the ability of *S. aureus* to form a biofilm, a biofilm assay was performed [93]. The *S. aureus* parent strains MW2 and JE2 were treated with SK-03-92 at concentrations of 0.5–64 µg/mL and those plates were compared to wells with bacteria not treated with the drug. After drying, the remaining dryed crystal violet dye stained biofilm material was extracted with 160 µL 33% glacial acetic acid per well and the OD_570_ was measured for each well. The total biofilm assay was performed a minimum of 10 times for each strain to achieve statistical significance.

### 3.9. MICs

In vitro minimum inhibitory concentration (MIC) determinations were performed on the *S. aureus* strains using SK-03-92 according to the Clinical and Laboratory Standards Institute guidelines [94]. All MICs were done a minimum of three times.

### 3.10. Statistical Analysis

A two-tailed Student′s *t*-test was run for the qRT-PCR comparisons and an ANOVA analysis was used for the biofilm assays to assess probabilities. *p*-values < 0.05 were considered significant.

## 4. Conclusions

Drug treatment with the stilbenoid compound SK-03-92 caused more genes to be transcriptionally downregulated than upregulated compared to other bactericidal and stilbenoid compounds (e.g., pterostilbene and resveratrol). The methoxy substitution on the main benzene ring at position 5 is likely to be responsible for this effect. A putative TCS, MW2284/MW2285, is clearly downregulated by SK-03-92 treatment. Is the TCS the prime target of the SK-03-92 lead compound and could targeting this TCS be the mechanism of action for SK-03-92 in Gram-positive bacteria? We hypothesize that one of the SK-03-92 targets is this putative TCS. Knockouts of both *MW2284* and *MW2285* showed substantial upregulation of the *srtA* gene that encodes sortase A. Sortase A may present something on the exterior of the *S. aureus* cell that causes rapid cell lysis. Furthermore, the MW2284 and MW2285 ORFs lie just upstream of the MW2286 ORF, which is thought to encode a malate:quinone oxidoreducatase gene important in the electron transport chain. If the MW2284/MW2285 TCS positively regulates this gene, then a mutation in either gene or treatment of *S. aureus* with a SK-03-92 drug may, in turn, cause downregulation of this gene as well as *sdhC* and *glpD* that would disrupt the electron transport chain in *S. aureus*. Evidence presented in this study also suggests the existence of a conserved bacterial pathway, involving PCD and persister formation, which is triggered by protein glycation and aggregation that may be responsible for the killing mechanism of SK-03-92. Could this putative TCS be tied to these phenomena? Further study may help us determine if the SK-03-92-induced *S. aureus* cell lysis is caused by a disruption of the electron transport chain, regulation of a conserved prokaryotic PCD pathway, or a combination of both of these events.

## Figures and Tables

**Table 1:** Bacterial strains used in this study.

Bacterial Strain	Genotype	Reference
*S. aureus*		
MW2	USA400 wild-type	[7]
JE2	USA300 wild-type	[38]
NE272	JE2 *MW2284* mutant	[38]
NE671	JE2 *MW2285* mutant	[38]
NE1363	JE2 *srtB* mutant	[38]
NE1787	JE2 *srtA* mutant	[38]
*L. monocytogenes*		
EGD	Wild-type	[39]
EGD *srtA*	EGD *srtA* mutant	[39]
EGD *srtB*	EGD *srtB * mutant	[39]

**Table 2:** Microarray analysis of genes dysregulated in *S. aureus* MW2 cells treated with 8× the SK-03-92 MIC vs. untreated cells.

Locus	Fold-Difference	Description
Stress Response		
SACOL1759	−2.3	universal stress protein family
Transporter		
SACOL0086	−2.0	drug transporter, putative
SACOL0155	−5.7	cation efflux family protein
SACOL0178	−2.9	PTS system, IIBC components (*scrBC*)
SACOL0400	−2.6	ascorbate-specific PTS system subunit IIC (*ulaA*)
SACOL0454	−2.3	sodium:dicarboxylate symporter family protein
SACOL1018	−2.3	sodium:alanine symporter family protein
SACOL1872	−3.0	epidermin immunity protein F (*epiE*)
SACOL2146	−2.7	PTS system, mannitol-specific IIBC components (*mtlA*)
SACOL2333	−2.8	YnfA family protein
SACOL2573	−3.2	copper ion binding protein (*copZ*)
SACOL2664	−2.3	mannose-6-phosphate isomerase (*manA*)
SACOL2718	−4.6	2-oxoglutarate/malate translocator, sodium sulfate symporter
Signaling/Regulation		
SACOL2360	−14.1	LytTR family regulator protein
SACOL2361	−26.9	histidine kinase sensor membrane protein
SACOL2340	2.2	transcriptional regulator TetR-family
Cell Wall Associated		
SACOL0151	−2.7	UDP-*N*-acetylglucosamine 2-epimerase Cap5P (*cap5P*)
SACOL0247	−3.2	holin-like protein LrgA (*lrgA*)
SACOL0612	−2.1	glycosyl transferase, group 1 family protein
SACOL1071	−2.2	chitinase-related protein (*iraE*)
SACOL2554	−2.0	holin-like protein CidB (*cidB*)
SACOL2539	4.2	sortase A (*srtA*)
Anabolism/Nucleic Acids		
SACOL013	−2.1	5′ nucleotidase family protein
SACOL1078	−3.2	phosphoribosylformylglycinamidine synthase II (*purL*)
SACOL1082	−2.5	bifunctional purine biosynthesis protein (*purH*)
SACOL1083	−2.6	phosphoribosylamine-glycine ligase (*purD*)
SACOL2329	−3.5	ribose 5-phosphate isomerase (*rpiA*)
SACOL2111	2.2	thymidine kinase (*tdk*)
SACOL2377	2.3	conserved hypothetical protein
Anabolism/Proteostasis		
SACOL0085	−2.5	peptidase, M20.M25/M40 family
SACOL2605	−9.6	ribulosamine 3-kinase
SACOL0457	2.6	conserved hypothetical protein, heat induced stress
SACOL0590	2.4	30S ribosomal protein L7 Ae
SACOL0877	2.5	glycine cleavage system H protein (*gcvH*)
SACOL1907	2.4	ribosomal large subunit pseudouridine synthase (*rluD*)
SACOL1939	2.3	phosphotyrosine protein phosphatase (*ptpA*)
SACOL2596	2.6	metallo-dependent amidohydrolase
Lipid Metabolism		
SACOL2091	−2.5	beta-hydroxyacyl-dehydratase FabZ (*fabZ*)
SACOL2459	−3.8	para-nitrobenzyl esterase (*pnbA*)
SACOL1142	10.0	aerobic glyerol-3-phosphate dehydrogenase (*glpD*)
Catabolism		
SACOL0135	−2.4	alcohol dehydrogenase, iron-containing (*adhE*)
SACOL0660	−3.4	alcohol dehydrogenase, zinc-containing (*adhA*)
SACOL1158	−2.5	succinate dehydrogenase, cytochrome b558 subunit (*sdhC*)
SACOL1604	−2.1	glucokinase (*glk*)
SACOL2338	−3.5	hypothetical protein (putative oxidoreductase)
SACOL1713	2.3	hypothetical protein, putative ammonia monooxygenase
Unknown		
SACOL0089	−4.4	myosin-reactive antigen, 67 kDa
SACOL2315	−3.8	conserved hypothetical protein
SACOL2338	−3.4	conserved hypothetical protein
SACOL2491	−2.9	conserved hypothetical protein
SACOL0742	3.1	conserved hypothetical protein
SACOL1789	2.4	conserved hypothetical protein

**Table 3:** MIC results for *S. aureus* and *L. monocytogenes* mutants and wild-type strains against SK-03-92.

Strain	Genotype	MIC
*S. aureus*		
JE2	Wild-type	1 ^a^
NE272	*MW2285*	1
NE671	*MW2284*	1
NE1363	*srtB*	1
NE1787	*srtA*	0.5
*L. monocytogenes*		
EGD	Wild-type	1
EGD *srtA*	*srtA*	0.125
EGD *srtB*	*srtB*	1

^a^ Mean + standard deviation from three separate runs.

**Table 4:** Oligonucleotide primers used in this study.

Primer	Gene	Sequence
SaFtsZ1	*ftsZ*	5′-GGTGTAGGTGGTGGCGGTAA-3′
SaFtsZ2		5′-TCATTGGCGTAGATTTGTC-3′
GuaBF1	*guaB*	5′-GCTCGTCAAGGTGGTTTAGGTG-3′
GuaBR1		5′-TAAGACATGCACACCTGCTTCG-3′
SrtA1	*srtA*	5′-TCGCTGGTGTGGTACTTATC-3′
SrtA2		5′-CAGGTGTTGCTGGTCCTGGA-3′
MW2284A	*MW2284*	5′-CAATGCAAATGAGACGGAATCT-3′
MW2284B		5′-GAAGAATAGGTGTAGTGTGCAT-3′
MW2285A	*MW2285*	5′-GTATGTTATTTGCAGACGGCAA-3′
MW2285B		5′-AAAGGCAAGAATCCGACATACG-3′
SA2043A	*tdk*	5′-CTTGTTCACTGACAGCCATCA-3′
SA2043B		5′-ACGCACGACTTAACTAATGTTG-3′
SaPurD1	*purD*	5′-CAGCCGCTAATTGATGGATTA-3′
SaPurD2		5′-AGCACTTCTGGCTGCTTCAAT-3′
SaPurH1	*purH*	5′-CCAGAAATAATGGATGGCCGT-3′
SaPurH2		5′-TGCCGGATGTACAATTGTTGT-3′
SaPurL1	*purL*	5′-GTTATGTGGAGTGAACATTGC-3′
SaPurL2		5′-AGCCCCAATAGAGACAATGTC-3′
